# Genomovirus Genomes Recovered from *Echinothrips americanus* Sampled in Florida, USA

**DOI:** 10.1128/genomeA.00445-17

**Published:** 2017-05-25

**Authors:** Simona Kraberger, Jane E. Polston, Heather M. Capobianco, Ricardo I. Alcalá-Briseño, Rafaela S. Fontenele, Arvind Varsani

**Affiliations:** a The Biodesign Center for Fundamental and Applied Microbiomics, Center for Evolution and Medicine, School of Life Sciences, Arizona State University, Tempe, Arizona, USA; b Department of Plant Pathology, University of Florida, Gainesville, Florida, USA; c Structural Biology Research Unit, Department of Clinical Laboratory Sciences, University of Cape Town, Observatory, Cape Town, South Africa

## Abstract

Four genomovirus genomes were recovered from thrips (*Echinothrips americanus*) collected in Florida, USA. These represent four new species which are members of the *Gemycircularvirus* (*n* = 2),* Gemyduguivirus* (*n* = 1), and *Gemykibivirus* (*n* = 1) genera. This is the first record, to our knowledge, of genomoviruses associated with a phytophagous insect.

## GENOME ANNOUNCEMENT

*Genomoviridae* is a recently established (1) but rapidly growing family of small circular single-stranded DNA (ssDNA) viruses comprised of nine genera (2). Genomoviruses have been recovered from a broad range of environments, organisms, and sample types. Seven species of genomoviruses have been associated with insects. These include five species within the *Gemycircularvirus* genus (accession numbers JX185429, HQ335086, KM598385 to KM598388, and KM598382 to KM598384), four of which were identified in dragonflies, one in both a dragonfly and a damselfly, and one from mosquitoes. Additionally, two species were recovered from dragonflies belonging to the *Gemyduguivirus* (accession no. JX185428) and *Gemykibivirus* (accession no. JX185430) genera (3–5). A recent study by Jiang et al. (6) demonstrated that the fungus-infecting genomovirus *Sclerotinia gemycircularvirus-1* is also able to replicate in insect cells, and thus, it is highly likely that some insect-associated genomoviruses identified to date may be actively circulating in insects.

Phytophagous insects play an important role in the spread of many plant viruses (7). As part of our efforts to identify viruses in phytophagous insects, we sampled adult thrips (species *Echinothrips americanus*) by aspiration from wild mustard, squash, and tomato plants from an agricultural field in Balm, FL, USA, in July 2014. Thrips were homogenized in 1 ml of SM buffer (0.1 M NaCl, 50 mM Tris-HCl [pH 7.4]) and centrifuged at 9,500 × *g* for 10 min, and the supernatant was filtered through a 0.2-µm syringe filter. This filtrate was subsequently used to extract total viral DNA using the High Pure viral nucleic acid kit (Roche Diagnostics, USA), and circular DNA was amplified using the Illustra TempliPhi kit (GE Healthcare, USA). The viral DNA from adult thrip samples was sequenced on an Illumina HiSeq 2000 platform at the Beijing Genomics Institute (Hong Kong). Paired-end reads from this data set were *de novo* assembled using ABySS 1.9 (8). Four contigs >1,000 nucleotides (nt) which shared similarity to known genomoviruses were identified using BLASTx (9), and back-to-back primers were designed based on these contig sequences. These specific primers were used to amplify the four viral genomes by PCR using Kapa HiFi HotStart DNA polymerase (Kapa Biosystems, USA), and amplicons were cloned and Sanger sequenced by primer walking (Macrogen, Inc., South Korea). The four genomovirus genomes (accession numbers KY308268 to KY308271) share <70% genome-wide nucleotide pairwise identity and have ~2.2-kb genomes. Two genomes, thrp_197969 (accession no. KY308268) and thrp_197934 (accession no. KY308271), are part of the *Gemycircularvirus* genus, sharing ~67% pairwise nucleotide identity with a genomovirus from equine feces (accession no. KT862248) and 82% sequence identity with a genomovirus from Pacific flying fox feces (accession no. KT732795), respectively, which were sampled in New Zealand. The third genome, thrp_197938 (accession no. KY308269), is the second member of the *Gemykibivirus* genus associated with an insect and shares 69% pairwise identity with a genomovirus from a dragonfly collected in Florida (accession no. JX185430). The fourth genome, thrp_197937 (accession no. KY308270), groups in the *Gemykolovirus* genus and is most closely related to isolates of a genomovirus from common bean in Brazil (accession numbers KX434768 to KX434770), sharing 68% nucleotide sequence identity. These four genomes complement the seven genomes that have been identified from insects (damselflies, dragonflies, and mosquitoes).

### Accession number(s).

The complete genome sequences of the genomovirus genomes obtained from thrips have been deposited at GenBank under accession numbers KY308268 to KY308271.
